# Antioxidant Capacity, Volatile Profile, and Physical Properties Changes of Kohlrabi Treated with Onion and Beetroot Juices Using Vacuum Impregnation Process

**DOI:** 10.3390/molecules30173563

**Published:** 2025-08-30

**Authors:** Magdalena Kręcisz, Marta Klemens, Joanna Kolniak-Ostek, Bogdan Stępień, Maciej Combrzyński, Aleks Latański

**Affiliations:** 1 Institute of Agricultural Engineering, Wrocław University of Environmental and Life Sciences, Chełmońskiego Street 37a, 51-630 Wrocław, Poland; 2 Department of Food Chemistry and Biocatalysis, Wrocław University of Environmental and Life Sciences, Norwida 25, 50-375 Wrocław, Poland; 3 Department of Fruit, Vegetable and Grain Technology, Wroclaw University of Environmental and Life Sciences, Chełmońskiego Street 37/41, 51-630 Wrocław, Poland; 4 Department of Food Process Engineering, University of Life Sciences in Lublin, Głęboka 31, 20-612 Lublin, Poland; maciej.combrzynski@up.lublin.pl

**Keywords:** kohlrabi, beetroot, onion, vacuum impregnation, volatile compounds, drying, antioxidant capacity, total phenolic content, texture

## Abstract

The aim of the study was to use vacuum impregnation (VI) with onion and beetroot juices as a pre-treatment before drying to develop innovative dried kohlrabi products. Two modern drying techniques were used: freeze-drying (FD) and vacuum drying (VD). The physicochemical properties were determined, including color, water activity, dry matter, density, volumetric gel index, texture, antioxidant capacity, total phenolic content (TPC), and volatile organic compounds (VOCs). It was shown that vacuum impregnation reduced the color lightness and springiness of kohlrabi. In addition, vegetables after VI showed an increase in dry matter, water activity, bulk density, volume gel index, color attributes a* and b*, color difference, hardness, and chewiness. Furthermore, the pre-treatment allowed for the introduction of additional VOCs characteristic of onions (1-Heptene, 2-methyl-(19.81%), Pentyl formate (19.81%), and 4-(Methylthio)butyl isothiocyanate (18.22%) in kohlrabi with onion juice: dimethyl trisulfide, methyl prop(en)yl disulfide, and 3,5-diethyl-1,2,4-trithiolane) and beetroot (dimethyl trisulfide), myrcene. The vacuum impregnation process significantly increased antioxidant capacity and total polyphenol content compared to raw samples. The results of dry weight, water activity, density, TPC, antioxidant capacity and texture in the case of freeze-dried products confirm that FD is a more advantageous method. In addition, freeze-drying allowed for significant preservation of volatile compounds and the color of kohlrabi. The results indicate the potential of VI as a method for modifying the properties of kohlrabi and producing functional and innovative dried products.

## 1. Introduction

Kohlrabi (*Brassica oleracea var. gongylodes*) belongs to the Cruciferae family (*Brassicaceae*), and its edible part is a thickened stem called a tuber. Kohlrabi belongs to the same species as cabbage, kale, broccoli, and cauliflower. The stem of kohlrabi can take on different colors, such as light green, white, or purple, depending on the variety [1,2,3].

This plant is widely cultivated in Europe and America, where it is popular due to its short growing season and favorable export potential, making it attractive to vegetable producers [4,5]. Kohlrabi is a vegetable widely used in the human diet, both raw and cooked. Its mild flavor and crunchy texture make it popular in various forms—in salads, raw, in soups, or as a side dish. Due to its slow rise in blood sugar levels and low calorie content, it is particularly recommended for people with diabetes, hypoglycemia, and atherosclerosis [6,7].

The thickened shoots, which constitute the main part of the plant, are juicy and have a characteristic, delicate flavor, especially when harvested in the early stages of development. Kohlrabi is characterized by high nutritional and health value, resulting from its rich content of vitamins (A, B1, B2, B5, B6, E), minerals (calcium, magnesium, zinc, and iron), and antioxidants, which may support cancer prevention [5,7,8,9]. Kohlrabi has anti-inflammatory and antioxidant properties due to its content of phenolic compounds, glucosinolates, and anthocyanins [3]. In addition, purple kohlrabi has significantly higher antioxidant and anti-diabetic properties than green kohlrabi because it contains higher levels of bioactive flavonoids, anthocyanins and phenolic compounds, which are important for humans [3,10]. Among the many cruciferous vegetables, kohlrabi has recently gained popularity and is widely consumed due to its pleasant taste. In addition, it is low in fat and carbohydrates [5,7].

A vegetable such as kohlrabi, with a moisture content of about 91%, is particularly susceptible to post-harvest losses and microbial degradation [4,11]. Literature data indicate that post-harvest losses of vegetables account for the largest percentage of all losses, ranging from 20 to 40% [4]. Drying is the most common method of food preservation. Its advantages include extending the shelf life of vegetables and fruits by inhibiting the growth of microorganisms [12,13]. Convection drying is still the most commonly used method of drying fruit and vegetables. However, this method causes a significant change in the physicochemical profile and sensory characteristics of the product [14]. Freeze-drying, also known as lyophilization, stands out as one of the most effective methods of preserving bioactive compounds. FD is the most important method among all drying methods, particularly due to the absence of high temperatures during drying. This process is based on the sublimation of ice under low pressure from previously frozen material [15,16]. An important advantage of freeze-drying is the minimization of density, water absorption capacity, reduction in physical, microbiological, and chemical changes, stabilization of cell structure, minimization of deformation and cracking, and significantly increased permeability. These parameters are achieved through the formation of ice crystals during freezing, which reconstruct the microstructure [15,17]. Although FD is considered the best method of food preservation, it is quite expensive [18]. Therefore, alternative methods are being sought, or process parameters are being changed in order to obtain the desired properties [15]. Vacuum drying is carried out at low temperatures and under reduced pressure. Thanks to this process, which is carried out in an oxygen-free environment, oxidation reactions, thermal degradation, and aromas are reduced, and less energy is consumed [19]. Literature data indicate that ginger extract and vacuum-dried honey fruits retained their bioactive compounds [15]. There is an increase in cardiovascular disease, type 2 diabetes, obesity, neurodegenerative diseases, and cancer among the general population. The main causes are considered to be the consumption of highly processed foods and a sedentary lifestyle [20,21]. Therefore, there is a growing demand for functional foods and beverages as people become more health conscious. They are looking for products that offer additional health benefits beyond the mere provision of nutrients, as well as gluten-free products [22,23].

Preliminary treatment of raw materials before drying allows for the proper preparation of the material intended for preservation. This enables the modification of bioactive properties, physical properties, texture, and volatile compounds [24,25,26,27]. Vacuum impregnation is considered a promising method for obtaining high-quality food products. In addition, this process improves the retention and transfer of beneficial phytochemicals, such as flavonoids, polyphenols, and chlorogenic acids. These substances are responsible for health-promoting and antioxidant properties. Literature data confirm that VI is an effective method for facilitating the infusion of bioactive compounds into the porous structure of coffee fruits [22]. VI has been used in matrices of materials such as sweet potatoes [27], cauliflower [25], zucchini and broccoli [26], cheese [28], fish [29], meat [30], and coffee fruit [22].

Several previous studies have been conducted on the properties of kohlrabi and its drying. Data on kohlrabi leaves are available in the literature, with studies including biochemical and physiological analyses, optical measurements, morphological measurements [2], and studies on the effect of flat covers and plant density on kohlrabi yield and quality [9]. Data confirming the anti-diabetic effect of kohlrabi are also available [10]. Most of the existing data focus on yields and nutrient content in tubers and leaves depending on the timing of spring transplanting [3], the study of the kinetics of the kohlrabi drying process, and thermal modeling for kohlrabi dried by the solar method [11].

There are no reports in the literature describing the effect of combining vacuum impregnation (VI) with onion and beetroot juice and freeze-drying (FD) and vacuum drying (VD) on the physical properties, volatile compounds, total phenolic content, antioxidant capacity of kohlrabi. The aim of this study was to use the above-mentioned methods to produce innovative and functional dried products and to describe their physical and chemical properties and volatile compounds. The results of this study will be particularly important for specialists involved in the development of innovative snacks or food additives with modified odor and antioxidant properties. In addition, knowledge of the physicochemical properties may help in the design of new types of innovative foods.

## 2. Results and Discussion

### 2.1. Analysis of the Volatile Compound Profile in Kohlrabi Samples with the Addition of Beetroot and Onion Juice

The volatile compound profiles were analyzed in kohlrabi prepared in three variants: pure (KO), with the addition of beetroot juice (KB), and with the addition of onion juice (KC). Each variant was tested in three forms: fresh, after freeze-drying (FD), and after vacuum drying (VD). The identification and determination of the relative percentage content of volatile compounds were carried out using the HS-SPME Arrow technique (GC-MS, Shimadzu QP 2020 Plus, Shimadzu, Kyoto, Japan).

In fresh kohlrabi (KO), the dominant volatile compounds were pentyl formate (19.81%) and 4-(methylthio)butyl isothiocyanate (18.22%) (Table 1). These compounds contribute significantly to the characteristic aroma of the vegetable. Pentyl formate is associated with a pleasant fruity scent [31], while 4-(methylthio)butyl isothiocyanate imparts the spicy and pungent odor typical of *Brassica* vegetables [32]. A noticeable variation in compound content was observed depending on the preservation method used. Freeze-drying (KO_FD) largely preserved the main aromatic compounds. Compared to the fresh sample, there was an increase in the proportion of pentyl formate, 1-heptene, and 2-methyl-, while the level of isothiocyanate decreased. In vacuum-dried samples (KO_VD), a general reduction in sulfur compounds was observed, suggesting their higher sensitivity to thermal and vacuum conditions.

The addition of beetroot juice significantly altered the volatile compound profile. In all KB samples, dimethyl trisulfide (Trisulfide <dimethyl->) was dominant, reaching as high as 61.07% in the fresh sample (KB), which imparted an intense, sulfurous, and even unpleasant odor to the sample.

Myrcene is one of the main aromatic compounds present in significant amounts after the addition of beetroot and onion juice to kohlrabi. However, during the drying process—both freeze-drying and vacuum drying—its content decreases significantly, which can be explained by its volatility and sensitivity to drying conditions, leading to its evaporation or chemical degradation.

A similar decrease is observed for dimethyl sulfide, which is responsible for a characteristic sulfurous aroma. Its level initially increases after the addition of beetroot or onion juice, but drying reduces its concentration, affecting the final aroma of the product.

On the other hand, many unidentified compounds introduced into kohlrabi with beetroot or onion juice increase in amount after the drying process. It is possible that chemical reactions occur during drying, leading to the formation of new substances or the concentration of less volatile compounds, resulting in their higher presence in the final product.

Both freeze-drying and vacuum drying significantly reduced the content of dimethyl trisulfide—to 53.59% and 10.14%, respectively—suggesting that vacuum drying may be an effective method to limit the intense sulfur aroma caused by the addition of beetroot juice.

The aroma profile of KO samples (with onion juice) was dominated by sulfur compounds typical of onion: dimethyl trisulfide (up to 17.17% in KO_FD), methyl prop(en)yl disulfide, and 3,5-diethyl-1,2,4-trithiolane—all characterized by strong, pungent odors. The addition of onion juice significantly enriched the profile with sharp, characteristic sulfur notes, which were most intense after freeze-drying. KO_FD samples showed the highest content of sulfur compounds, while vacuum-dried samples (KO_VD) exhibited lower concentrations, similarly to KB_VD [33].

The addition of beetroot and onion juice significantly modified the volatile compound profile in kohlrabi. The beetroot juice variant was characterized by a dominant presence of dimethyl trisulfide, responsible for an intense sulfurous odor that may be undesirable from a sensory perspective. In contrast, the addition of onion juice introduced a more diverse set of sulfur compounds, giving the samples a spicy, onion-like character. In pure kohlrabi samples, typical *Brassica*-derived compounds, such as pentyl formate and isothiocyanates, were dominant. The formation of unidentified volatile compounds in the enriched samples suggests potential interactions between components during processing.

The applied drying method clearly affected the aroma profile. Freeze-drying allowed for better preservation of volatile compounds, especially sulfur-containing ones, resulting in a more intense aromatic profile, particularly in samples enriched with onion juice. Vacuum drying reduced the content of sulfur volatiles, which may be beneficial in mitigating sharp and potentially off-putting aroma notes. These findings confirm that both the selection of vegetable juices and the preservation technology are effective tools for shaping the sensory properties of kohlrabi-based products.

**Figure 1:**
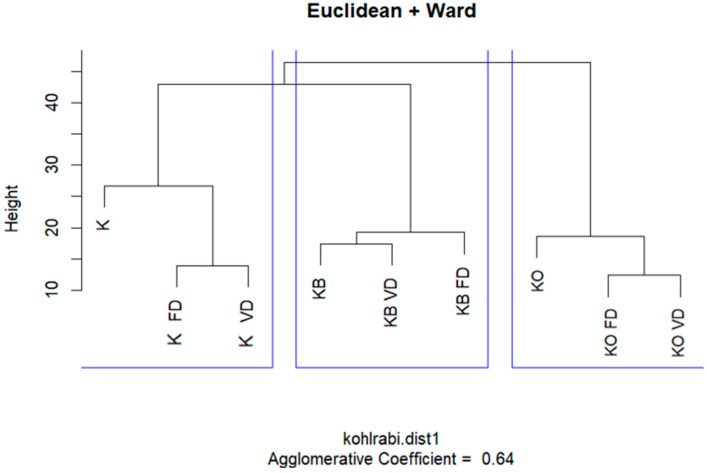
HCA result analysis of fresh and dried kohlrabi with beetroot and onion juice.

The dendrogram generated through hierarchical cluster analysis (using Euclidean distance and Ward’s method) clearly distinguished three main groups of kohlrabi samples based on their volatile compound profiles (Figure 1). The first group included the pure kohlrabi samples—fresh, freeze-dried (KO_FD), and vacuum-dried (KO_VD)—whose close clustering suggests that the drying method did not significantly alter the volatile profile in the absence of juice additives. The second group comprised samples with beetroot juice (KB, KB FD, KB VD), which also formed a cohesive cluster regardless of processing method. A similar pattern was observed in the third group—kohlrabi with onion juice (KO, KO_FD, KO_VD)—where no clear differences were evident due to the type of thermal processing.

These results indicate that the type of additive (beetroot or onion juice), rather than the processing method (freeze-drying or vacuum drying), is the primary factor differentiating the samples in terms of volatile compound profiles. This suggests that volatile compounds originating from the added juices play a key role in determining the final aroma characteristics of the product, regardless of the preservation method used.

The results of the volatile compound profile analysis in kohlrabi indicate that significant aroma changes arise from both the drying method and the addition of vegetable juices (beetroot and onion). In this study, the additives played a key role—especially onion juice, which introduced characteristic sulfur compounds, resulting in a distinctly different aroma profile. Similar findings were reported in our previous research on zucchini impregnated with onion juice, kale juice, and a mixture of both [34]. However, this effect is not always dominant—whether drying method or juice impregnation has a greater impact on the final aroma profile depends on the type of vegetable, its cellular structure, water content, and the reactivity of its native constituents [26]. Kohlrabi, with its relatively loose structure and high water content, may allow easier migration of compounds from added juices, making their influence more pronounced than in root or leafy vegetables.

At the same time, the drying method plays a crucial role in preserving or degrading volatile compounds—for instance, freeze-drying and vacuum drying are more effective at retaining volatile compounds compared to other drying techniques, especially in the context of fruits and vegetables [35,36]. Additionally, as highlighted by Xue et al. (2020), the choice of drying method is critical not only for preserving aroma-active volatiles but also for maintaining the stability of bioactive compounds and the overall quality of dried foods, which makes this technological decision particularly important in the development of functional vegetable-based products [37]. Ultimately, the final outcome is the result of interactions between the raw material’s structure, its native chemical composition, the applied additive, and the drying conditions. As demonstrated by Okonkwo et al. (2024), there is no universal rule for what determines the aroma of a dried product—each vegetable type must be analyzed individually [38].

Changes in the aromatic compound profile of kohlrabi (*Brassica oleracea*) during technological processing may result from several overlapping physicochemical and biochemical mechanisms. During freeze-drying and vacuum drying, partial loss of volatile aroma compounds occurs due to their evaporation along with moisture removal, despite the relatively mild thermal conditions of these processes [39]. In addition, cutting and processing of plant material induce stress responses that activate enzymes such as myrosinase and lipoxygenase, leading to the degradation of glucosinolates and the formation of sulfur-containing compounds with an intense, characteristic aroma [40]. Furthermore, impregnation with beetroot or onion juice results in the diffusion of exogenous secondary metabolites (including thiosulfinates) into the kohlrabi tissue, significantly modifying its aromatic profile by enriching it with earthy or sulfurous notes [25]. As a result, the final product exhibits clearly altered sensory properties, driven by the synergistic effects of physical removal, enzymatic transformation, and chemical enrichment of aroma compounds. Our results confirm that achieving the desired sensory profile requires careful selection of raw materials, additives, and an appropriately matched drying method, as each of these factors may influence the final composition of volatile compounds in different ways.

### 2.2. Dry Matter (DM) for Fresh Kohlrabi and After the Vacuum Impregnation Process

**Figure 2:**
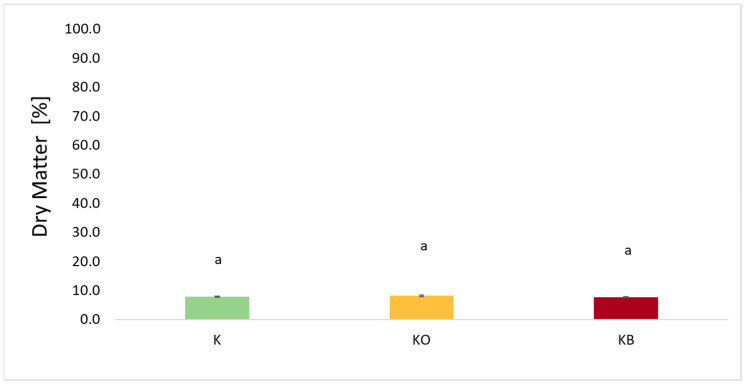
The effect of the vacuum impregnation process on the dry matter content in fresh kohlrabi (K), kohlrabi after VI with onion juice (KO), and kohlrabi after VI with beetroot juice (KB). The values marked with different lowercase letters indicate significant differences (*p* < 0.05).

Figure 2 shows the dry matter content of fresh kohlrabi (K) that has not undergone vacuum impregnation and kohlrabi after vacuum impregnation with various impregnating solutions, i.e., onion juice (O) and beetroot juice (B). The dry matter content of fresh kohlrabi was 7.86%. Depending on the impregnation solution used, the DM values ranged from 8.19% for KO to 7.60% for KB. In studies where onion juice was used as an impregnation solution, an increase in dry matter content was observed in cauliflower [25] and sweet potato [27]. Similarly, for beetroot juice, an increase in dry matter content was observed in cauliflower [25] and celery [41]. The opposite relationship, i.e., a decrease in dry matter, was observed in the case of zucchini which was subjected to the VI process using sodium chloride [34].

### 2.3. Dry Matter (DM) for Kohlrabi After the Drying Process

**Figure 3:**
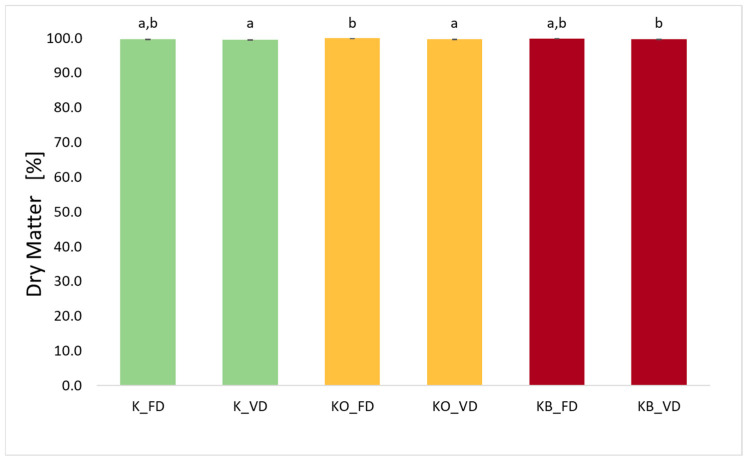
The effect of the vacuum impregnation process on the dry matter content in dried kohlrabi (K), kohlrabi after VI with onion juice (KO), and kohlrabi after VI with beetroot juice (KB). FD—freeze-drying, VD—vacuum drying. The values marked with different lowercase letters indicate significant differences (*p* < 0.05).

The dry matter content of kohlrabi after freeze-drying (FD) and vacuum drying (VD) is presented in Figure 3. The dry matter values range from 99.51% to 99.97%. Statistical analysis showed a significant effect of the drying method on the value of the tested parameter (*p* = 0.049). Higher DM values were observed for kohlrabi dried by the freeze-drying method. Other researchers also reported the highest dry matter values (or lowest moisture values) after freeze-drying when studying strawberries [14] and sweet potatoes [27]. This is because during freeze-drying, the structure and shape of the food are preserved, creating a larger diffusion area, which leads to further reduction in moisture in the food [14]. The study found that the use of both onion juice and beetroot juice increases the dry matter content in the dried material. It was noted that VI is statistically significant (*p* = 0.03).

Overall, statistical analysis revealed a strong negative correlation between DM and density (r = −0.897) and AW (r = −0.997). As well as a moderate negative correlation between ABTS (r = −0.685), FRAP (r = −0.570), and DPPH (r = −0.619).

### 2.4. Water Activity (AW) of Fresh Kohlrabi, Dried Kohlrabi and Before and After Vacuum Impregnation

**Figure 4:**
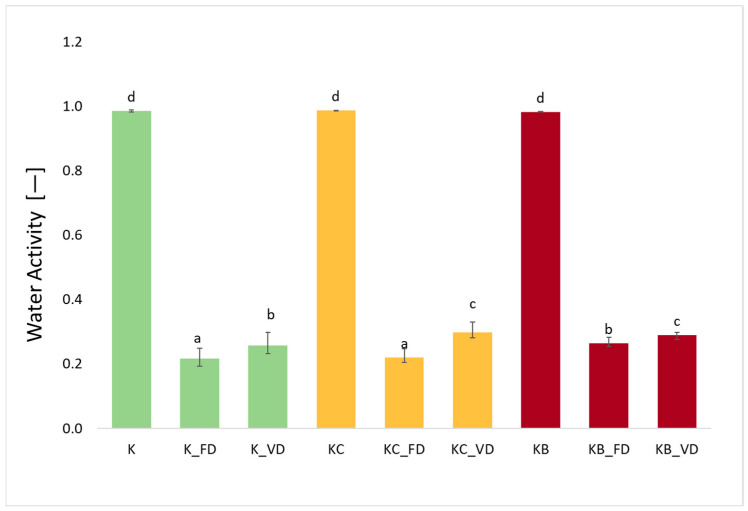
The effect of the vacuum impregnation process and drying method on the water activity in kohlrabi (K), kohlrabi after VI with onion juice (KO), and kohlrabi after VI with beetroot juice (KB). FD—freeze-drying, VD—vacuum drying. The values marked with different lowercase letters indicate significant differences (*p* < 0.05).

The water activity for fresh materials not subjected to drying processes was 0.985 (K), 0.986 (KO), and 0.982 (KB), respectively (Figure 4). The use of different drying methods was statistically significant (*p* = 0.00). The lowest AW values were obtained for materials dried by the freeze-drying method. The lowest water activity in materials after FD, compared to other drying techniques, was also obtained for sweet potatoes [27], cauliflower [25], pumpkin [42], and mushrooms [43]. In materials after vacuum impregnation dried by freeze-drying and vacuum drying, higher water activity was recorded than in kohlrabi without VI. Similar observations were made for cauliflower after impregnation with onion juice and beetroot juice [25]. However, the opposite relationship was observed for sweet potatoes after impregnation with onion juice and kale juice [27], as well as for zucchini after impregnation with beetroot juice [26]. Despite a clear increase in AW in dried products after VI, the use of pre-treatment was not statistically significant (*p* = 77). Moreover, AW correlated moderately positively with ABTS (r = 0.678), DPPH (r = 0.609), FRAP (r = 0.552), and density (r = 0.919), and strongly and negatively with DM (r = −0.997).

### 2.5. Bulk Density (ρ) of Fresh Kohlrabi, Dried Kohlrabi and Before and After Vacuum Impregnation

**Figure 5:**
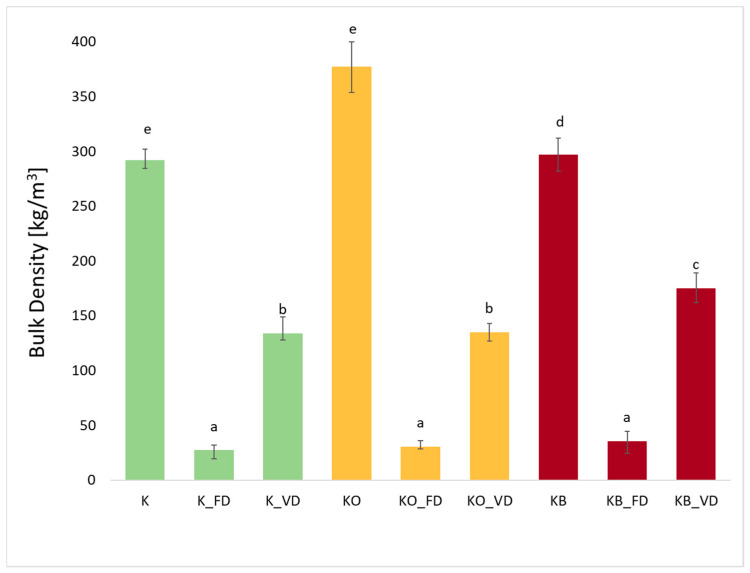
The effect of the vacuum impregnation process and drying method on the bulk density in kohlrabi (K), kohlrabi after VI with onion juice (KO), and kohlrabi after VI with beetroot juice (KB). FD—freeze-drying, VD—vacuum drying. The values marked with different lowercase letters indicate significant differences (*p* < 0.05).

The bulk density of kohlrabi after freeze-drying (FD) and vacuum drying (VD) is shown in Figure 5. The use of vacuum impregnation did not significantly affect the value of the tested parameter (*p* = 0.88). The density of fresh kohlrabi was 290.00 kg/m^3^, while the density of kohlrabi after vacuum impregnation with onion juice (KO) was the highest and amounted to 377.38 kg/m^3^. A slightly lower density in bulk was observed in fresh kohlrabi after vacuum impregnation with beetroot juice (KB) at 296.88 kg/m^3^. Other researchers have also observed an increase in bulk density after vacuum impregnation in cauliflower [25] and broccoli after impregnation with beetroot juice [44]. In the material after VI, as well as after freeze-drying and vacuum drying, higher values of bulk density were also observed compared to the fresh material without VI. Considering the material after impregnation and drying with SS and VD, kohlrabi with beetroot juice obtained higher values. Kohlrabi after vacuum drying exhibited lower values than fresh kohlrabi, but higher values than kohlrabi after SS, which is related to the specificity of the individual methods. Density after VD was 133.79 (KO_VD), 134.78 (KC_VD), and 174.90 (KB_VD), respectively. It can be observed that the use of different drying techniques had a significant effect (*p* = 0.00) on the reduction in bulk density. Freeze-dried kohlrabi had the lowest bulk density values, which were 27.28 kg/m^3^ (K_FD), 30.42 kg/m^3^ (KO_FD), and 35.71 kg/m^3^ (KB_FD), respectively. It is clear that the use of freeze-drying significantly reduced bulk density. Shams et al. compared the properties of mushrooms after freeze-drying and cabinet drying. The results showed that FD mushrooms had the lowest density [43]. Similar results were obtained by the authors of this study in earlier research on cauliflower impregnated with onion juice and beetroot juice [25], as well as zucchini and broccoli impregnated with beetroot juice [26].

### 2.6. Volumetric Gel Index (VGI) of Dried Kohlrabi and Before and After Vacuum Impregnation

**Figure 6:**
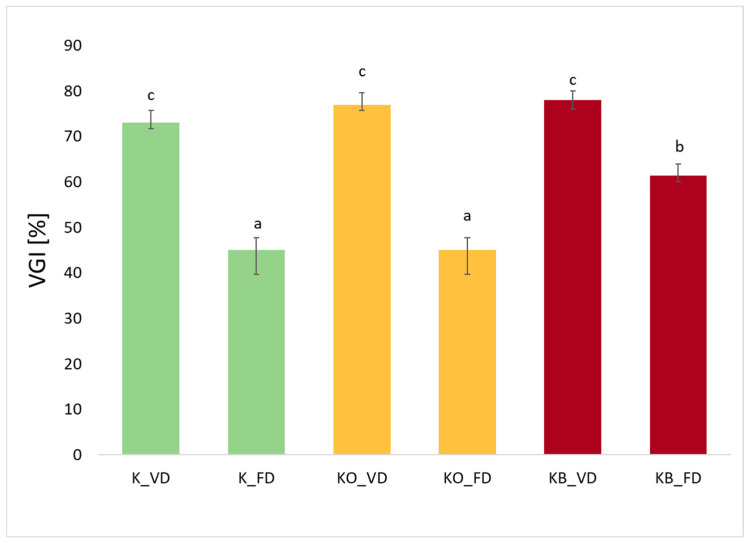
The effect of the vacuum impregnation process and drying method on the Volumetric Gel Index in kohlrabi (K), kohlrabi after VI with onion juice (KO), and kohlrabi after VI with beetroot juice (KB). FD—freeze-drying, VD—vacuum drying. The values marked with different lowercase letters indicate significant differences (*p* < 0.05).

Analyzing Figure 6, which shows the volume gelation index for the tested kohlrabi samples, we can see that these values range from 45% to 78%. The highest values were recorded for kohlrabi after vacuum drying, which were 73%, 77%, and 78% for K_VD, KC_VD, and KB_VD, respectively. A significant decrease in the value of the tested index can be observed in the case of freeze-drying, which was 45%, 45%, and 61% for K_FD, KC_FD, and KB_FD, respectively. These results are consistent with our previous studies, in which VGI was tested for broccoli and zucchini after FD and VD [26]. This is due to the fact that freeze-drying preserves a more porous, open structure of the material, which facilitates rapid water absorption during rehydration but leads to the formation of a less compact gel and lower VGI values [26,45]. Statistical analysis showed a significant effect of the drying method on the value of the tested parameter (*p* = 0.00). An increase in the value of the tested index was observed in materials after vacuum impregnation. This is particularly evident in kohlrabi after the application of beetroot juice after FD and VD. In broccoli and zucchini after vacuum impregnation with beetroot juice, higher VGI values were also observed compared to the control sample [26]. The use of VI in these studies showed a statistically significant effect of the vacuum impregnation process on VGI values (*p* = 0.04). Taking into account fresh kohlrabi (K) and after the vacuum impregnation process using beetroot and onion juice, higher VGI values were observed in the materials after VI. Of the two tested impregnation solutions, beetroot juice was the one that caused slightly higher values of the tested parameter than onion juice.

### 2.7. Color of Dried Kohlrabi and Before and After Vacuum Impregnation

**Figure 7:**
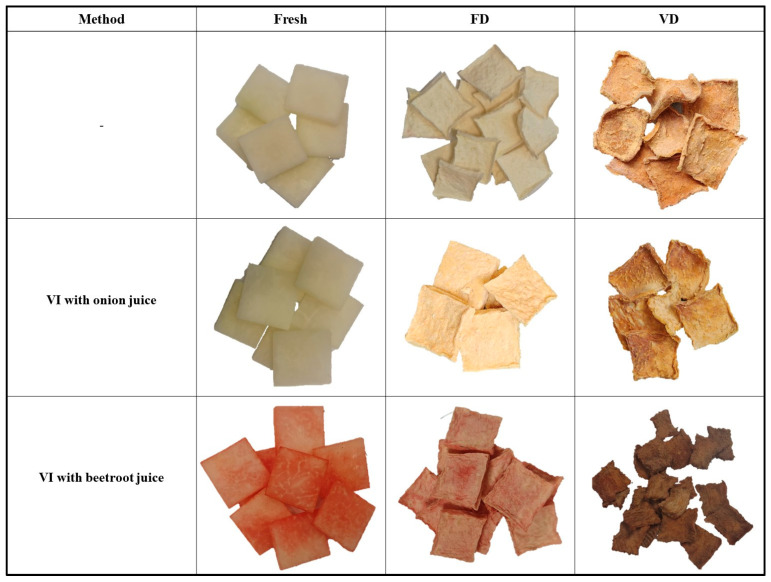
Visual comparison of selected kohlrabi pieces subjected to vacuum impregnation (VI) using onion and beetroot juice. FD—freeze-drying; VD—vacuum drying.

Table 2 presents color changes observed in fresh kohlrabi (K), after vacuum impregnation with onion juice (KO), after vacuum impregnation with beetroot juice (KB), and after freeze-drying (FD) and vacuum drying (VD). The results showed statistically significant differences in L* (lightness) depending on the drying method (*p* = 0.00) and the vacuum impregnation process (*p* = 0.00). Among all the materials tested, the highest values were recorded for kohlrabi after freeze-drying, which were 92.50 (K_FD), 91.23 (KO_FD) and 75.72 (KB_FD), respectively. Other researchers who subjected kohlrabi to freeze-drying also recorded the highest lightness values (92.50) [46]. Furthermore, in studies on melon [43,47] and cauliflower [25], the highest lightness values were also observed for freeze-dried materials. The use of vacuum impregnation and various impregnating solutions significantly affected the lightness values, causing a decrease in the tested parameter. Figure 7 shows a visual comparison of selected pieces of kohlrabi in all tested combinations. In addition to a significant change in color, it can be seen that the materials after freeze-drying have the best shape retention. This is due to the formation of ice crystals in the material, which better preserve the microstructure [17]. Kohlrabi after vacuum drying, on the other hand, is characterized by significantly greater shape deformation. This phenomenon is typical for materials dried in an oven and by natural methods [17]. The lowest lightness values were observed in kohlrabi samples after VI with beetroot juice. This phenomenon can be attributed to the dark and intense color of the impregnating solution. Other studies confirm this phenomenon. Authors who investigated the effect of vacuum impregnation with beetroot juice reported a decrease in lightness in broccoli [26,44], zucchini [26], celery [41], and cauliflower [25]. In the literature data cited above, brightness decreases in varying degrees. The smallest differences in brightness change were observed in broccoli, which is naturally darker in color [26]. Kohlrabi dried under vacuum at 60 °C was darker in color than after freeze-drying. In this case, the color of kohlrabi depended primarily on the Maillard reaction and caramelization. These are phenomena that occur in materials dried at elevated temperatures and contribute to the formation of brown pigment on the surface of the tested materials [48].

The drying method had little effect on the a* values (negative—green hue, positive—red hue) [49], although this effect was statistically significant (*p* = 0.00). Significantly higher differences in the a* parameter were observed in fresh and impregnated materials. In the case of fresh kohlrabi and kohlrabi treated with onion juice, a green hue was observed. However, the use of beetroot juice resulted in a significant increase in the tested color attribute. VI significantly affected the a* values (*p* = 0.00).

In the present study, positive values of the b* color attribute were observed, indicating a yellow hue [48]. Both the drying method (*p* = 0.00) and the use of pre-treatment (*p* = 0.00) significantly affected the b* parameter values. The lowest values of the yellow hue were observed in fresh material and after VI. The use of the drying process increased the yellow color in the tested materials. The highest values were recorded in vacuum-dried samples. These observations are consistent with our other studies in which cauliflower was dried using the vacuum method [25]. Shams et al., studying the effect of freeze-drying and cabinet drying, observed a similar trend for parameters a and b. Namely, the values of color attributes a* and b* were lowest for freeze-dried mushrooms [43].

The lowest color difference values were recorded in kohlrabi after vacuum impregnation with onion juice (6.69). As expected, the highest ∆E values were recorded for all materials with the addition of beetroot juice. This relationship has also been confirmed by other researchers who used beetroot juice for impregnation: broccoli [26,44], zucchini [26], celery [41], and cauliflower [25].

### 2.8. Texture Profile Analysis (TPA) of Dried Kohlrabi and Before and After Vacuum Impregnation

**Figure 8:**
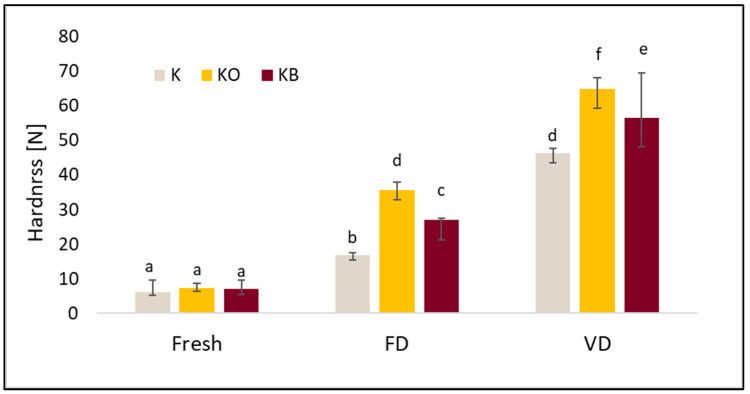
The effect of vacuum impregnation on the hardness of kohlrabi (K), kohlrabi after VI with onion juice (KO), and kohlrabi after VI with beetroot juice (KB). FD—freeze-drying, VD—vacuum drying. The values marked with different lowercase letters indicate significant differences (*p* < 0.05).

**Figure 9:**
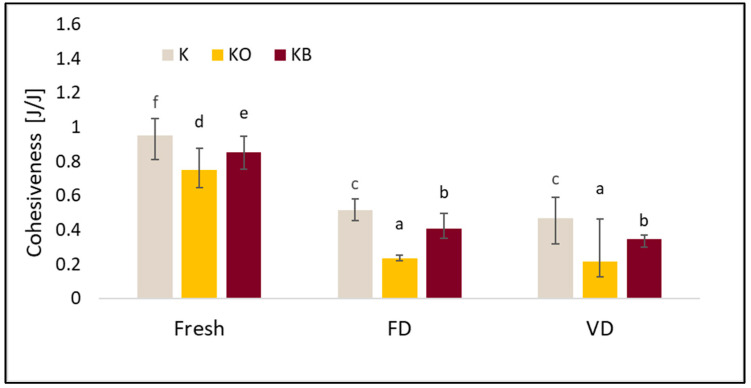
The effect of vacuum impregnation on the cohesiveness of kohlrabi (K), kohlrabi after VI with onion juice (KO), and kohlrabi after VI with beetroot juice (KB). FD—freeze-drying, VD—vacuum drying. The values marked with different lowercase letters indicate significant differences (*p* < 0.05).

**Figure 10:**
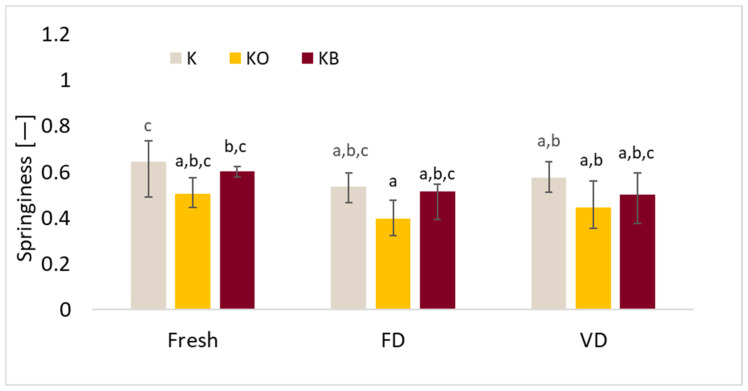
The effect of vacuum impregnation on the springiness of kohlrabi (K), kohlrabi after VI with onion juice (KO), and kohlrabi after VI with beetroot juice (KB). FD—freeze-drying, VD—vacuum drying. The values marked with different lowercase letters indicate significant differences (*p* < 0.05).

**Figure 11:**
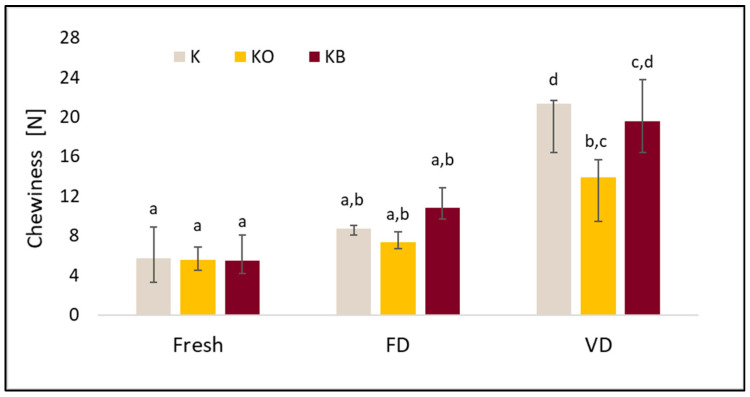
The effect of vacuum impregnation on the chewiness of kohlrabi (K), kohlrabi after VI with onion juice (KO), and kohlrabi after VI with beetroot juice (KB). FD—freeze-drying, VD—vacuum drying. The values marked with different lowercase letters indicate significant differences (*p* < 0.05).

The basic properties of the TPA test, such as hardness, cohesiveness, springiness, and chewiness, differed depending on the drying method and the vacuum impregnation process (VI). A detailed analysis of the TPA is presented in Figure 8, Figure 9, Figure 10 and Figure 11.

The hardness test shows that the use of drying and different drying methods increased the tested index. The highest hardness was obtained for kohlrabi after vacuum drying, with values of 46.31 N (K_VD), 64.80 N (KO_VD) and 56.59 N (KB_VD) (Figure 8). Significantly lower values were obtained for FD: 16.84 N (K_FD), 35.54 N (KO_FD), 26.93 N (KB_FD). We observed a similar trend in our previous studies [25]. Furthermore, statistical analysis confirmed that drying significantly affected the tested parameter (*p* = 0.00). Fresh kohlrabi was characterized by a hardness of 6.04 N. The use of pre-treatment significantly affected the tested parameter (*p* = 0.001). The greatest changes were observed in dried samples, as shown in the graph. The highest values were obtained for kohlrabi after VI with onion juice. These observations are consistent with other studies in which cauliflower was impregnated. The results of these studies indicate that the use of onion juice leads to the highest hardness, which may be due to the onion particles contained in the juice [25]. There was a strong negative correlation between hardness and cohesiveness (r = −0.844) and a moderate negative correlation with springiness (r = −0.555). Furthermore, a strong positive correlation was observed between hardness and gumminess (r = 0.813). No correlation was observed between hardness and VI (r = 0.137). Statistical analysis revealed a very strong positive correlation (r = 0.949) between hardness and the tested material (fresh, freeze-dried, and vacuum-dried).

Similarly to hardness, cohesiveness was significantly affected by the drying process (*p* = 0.00) and vacuum impregnation (*p* = 0.01). Fresh kohlrabi had the highest cohesiveness values (Figure 9). Drying caused a decrease in this parameter. The lowest cohesiveness values were obtained after vacuum drying. The use of VI caused a decrease in cohesiveness. The greatest differences were observed for kohlrabi after VI with onion juice, which was observed both in the material before and after drying. Statistical analysis showed a strong negative correlation between cohesiveness and fresh and dried material (r = −0.824) and a strong positive correlation between cohesiveness and elasticity (r = 0.836).

The results of the conducted tests showed that the drying process had a significant effect on the springiness results (*p* = 0.01), after which reduced values of the tested parameter were observed (Figure 10). It was demonstrated that changing the impregnating solution during the vacuum impregnation process did not affect the significance of the tested parameter (*p* = 0.85). The highest values of the studied parameter were recorded for fresh kohlrabi, which is consistent with previous studies in which we tested cauliflower. Despite this, the lowest springiness values were observed for kohlrabi after VI with onion juice. Similarly to our previous studies in which we investigated the effect of onion juice and beetroot juice on the properties of cauliflower [25].

In these studies, a significant effect of drying (*p* = 0.0000) on chewiness was observed. The lowest values of this parameter were recorded for fresh material, followed by freeze-dried material, and the highest for vacuum-dried material. The use of VI did not significantly affect the tested parameter (*p* = 0.91). Other authors observed the lowest values of the tested parameter in the case of the sublimation method, these authors studied *Pyracantha fortuneana* [50] and cauliflower [25].

### 2.9. Total Phenolic Content (TPC) and Antioxidant Capacity of Dried Kohlrabi and Before and After Vacuum Impregnation

Table 3 shows the results of the total phenolic content (TPC) and antioxidant capacity (ABTS, FRAP, DPPH) of fresh and dried kohlrabi samples. The phenolic content of the fresh kohlrabi analysed in this study was 339.51 mgGAE/100 g DM. TPC after vacuum impregnation was 603.62 mgGAE/100 g DM for KO and 588.12 mgGAE/100 g DM for KB, respectively. The use of vegetable juices significantly affected TPC in fresh and dried materials. Higher values of the studied parameter were observed in kohlrabi after impregnation with onion juice. In studies examining the effect of vacuum impregnation with beetroot juice on the properties of broccoli, an increase in TPC from 9.82 to 13.06 mgGAE/100 g was observed [44]. The highest phenolic contents, amounting to 958.59 mgGAE/100 g (KC_FD), 679.89 mgGAE/100 g (KB_FD), and 550.13 mgGAE/100 g (K_FD), were observed in the dried materials after freeze-drying. These values were from 30.52% to 100.87% higher compared to the phenolic contents of the materials after vacuum drying.

Higher TPC values for FD were also recorded when examining mushrooms [43]. Lower TPC values in the dried materials after VD can be attributed to exposure of the samples to higher temperatures and the occurrence of enzymatic reactions [43,51]. Other authors reported that ice crystals form in the frozen materials during freeze-drying, which enable increased extraction of phenolic compounds and easy access of solvents, leading to better TPC preservation [52].

The effect of vacuum impregnation, freeze-drying, and vacuum drying on the antioxidant capacity of kohlrabi is presented in Table 3. These studies were conducted using the ABTS, FRAP, and DPPH methods, with strong correlations observed. The studies indicate that pretreatment with onion juice and beetroot juice increases antioxidant capacity. The highest values of the studied parameter were recorded in kohlrabi after vacuum impregnation with onion juice, which was 2148.57 µMol Trolox/100 g of dry matter. In our other studies, in which we examined the properties of sweet potatoes, we observed the highest antioxidant activity values for materials after VI with onion juice and onion and kale juice [27]. Overall, drying resulted in a decrease in antioxidant activity. The method that best preserved the antioxidant properties of kohlrabi was the freeze-drying method. These results are consistent with other studies examining sweet potato [27], celery [35], melon peels [47], and mushroom powder [43].

### 2.10. PCA

**Figure 12:**
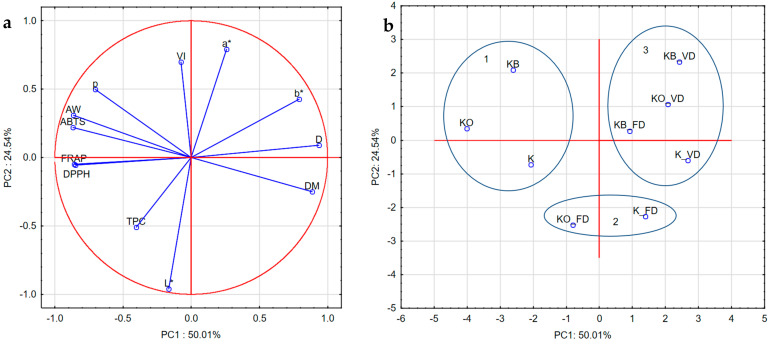
Loading plot (**a**) and score plot (**b**) of the principal component analysis (PC1 and PC2) carried out for dried kohlrabi. VI: vacuum impregnation; D: drying methods; DM: dry matter, AW: water activity; L*: lightness; a*, b*: parameters color; p: bulk density; ABTS, DPPH, and FRAP: antioxidant capacity; TPC: total phenolic content; FD: freeze-drying, VD: vacuum drying, 1,2,3: groups of parameters with similar characteristics.

Principal component analysis (PCA) was performed to identify differences in kohlrabi before vacuum impregnation, after vacuum impregnation with beetroot and onion juice, and after freeze-drying and vacuum drying (Figure 12a,b). PCA shows that the first component, PC1, explains 51.45% of the variability in the results and is negatively associated with VI, FRAP, DPPH, ABTS, AW, ρ, L*, and TPC. However, it was positively associated with a*, b*, and DM. The second principal component explains 26.63% of the results and is positively associated with VI, ρ, AW, ABTS, a*, and b*. However, it was negatively associated with DPPH, FRAP, TPC, L*, and DM. There was a strong but negative correlation between DM and water activity and density, and a moderate negative correlation with ABTS, FRAP, and DPPH. A moderate positive correlation was determined between ABTS, FRAP, DPPH and AW, ρ, and TPC, and a moderate negative correlation with DM, b*. There was a strong correlation between ABTS, FRAP, and DPPH. The color parameters a* and b* negatively correlated with L*, TPC, ABTS, FRAP, and DPPH.

Figure 12b shows the locations of kohlrabi samples. Close distances indicate similar characteristics, while larger distances indicate differences. The graph clearly shows that the samples differ from each other due to the vacuum impregnation process and drying methods used. PC1 shows positive values for kohlrabi after vacuum and freeze-drying, with the exception of sample KO_FD, whose characteristics are similar to those of the fresh samples. PC2, on the other hand, shows negative values for kohlrabi without pretreatment and positive values for kohlrabi after VI with onion juice and beetroot juice. Furthermore, three groups with similar characteristics are visible. The first group includes materials before heat treatment (K, KO, KB); the second group includes caperpa without VI and after VI with onion juice after freeze-drying; and the last group includes kohlrabi after vacuum drying and kohlrabi after VI with beetroot juice after FD.

## 3. Materials and Methods

### 3.1. Preparation of Sample

The subject of the study was kohlrabi, a low-calorie vegetable rich in nutrients, often used in the human diet. It is widely cultivated in Europe due to its flavor and health benefits, high yield, low temperature requirements, and short growing season, which allows for cultivation at various times during the season [3,4,6,7]. The raw material, of Polish origin, was obtained from the local vegetable market and, after transport to the laboratory, was stored under refrigeration conditions in a refrigerator ensuring appropriate temperature conditions (4 ± 2 °C). The raw material was stored to maximize the preservation of the material’s properties and freshness and to prevent adverse chemical and biological changes. Preparation of the root for testing included cleaning the leaves and stems, washing, drying, and peeling. Samples, prepared in the form of cuboids measuring 25 × 25 × 5 mm, were pretreated prior to drying by vacuum impregnation.

### 3.2. Pretreatment Before Drying Process

Vacuum impregnation of cuboidal kohlrabi cubes was performed using a prototype installation designed and constructed at the Institute of Agricultural Engineering, Wrocław University of Environmental and Life Sciences. Details of the installation’s construction and operation have been described in previous publications [41]. Vacuum impregnation is often used as an alternative to osmotic dehydration, causing structural changes within cell walls, which significantly impacts the drying process and the quality of the resulting dried material. The conditions for the pretreatment were determined based on literature data and the authors’ previous experience, adopting the most favorable parameters for the given raw material. The impregnation process took place in a stainless steel chamber, in which samples weighing 200 ± 2 g were placed. The vacuum impregnation process was carried out at a temperature of 22 °C. Freshly squeezed onion juice (11.5 ºBx) and organic red beetroot juice (7.5 ºBx) with an energy value of 155 kJ/37 kcal (Haus Rabenhorst, Unkel, Germany) were used for impregnation. Sample codes and detailed explanations are presented in Table 4. The main phases of the process were as follows: 4 in reduced pressure of 0.06 MPa, immersion of kohlrabi samples in one liter of impregnation solution for 4 min, and restoration of atmospheric pressure for 15 min. The impregnation conditions were crucial for achieving stabilization of the raw material and consolidating the effects of the pretreatment.

### 3.3. Drying

Two modern drying techniques were used to remove water from pretreated kohlrabi: freeze-drying (FD) and vacuum drying (VD). Both methods have well-understood theoretical foundations and hold great promise for producing high-quality dried products. Process parameters were adopted based on literature analysis to exclude drying under extreme conditions. To obtain a complete picture of the impact of vacuum impregnation as a pretreatment before drying on product quality, unimpregnated kohlrabi was also dried.

#### 3.3.1. Freeze Drying (FD)

Kohlrabi was frozen outside the dryer to eliminate the undesirable effect of self-freezing. An RL58GRGIH freezer (Samsung Electronics Polska sp. z o.o., Wronki, Poland) was used. Freezing conditions were as follows: temperature −18 °C, process duration 24 h at 5 Pa, and freezing rate 1° C min^−1^. Freeze-drying was performed in a Free-Zone 4.5 L system (Labconco, Fort Scott, KS, USA). Literature reports indicate that high hot plate temperatures and contact heat delivery to samples accelerate the water removal process but also deteriorate the quality of the dried product compared to products obtained at low process temperatures and with radiative heat delivery. This study was conducted using a compromise solution, which involved the use of contact heat delivery at a relatively low hot plate temperature of 22 °C and a heating chamber temperature of −50 °C.

#### 3.3.2. Vacuum Drying (VD)

The research presented in this paper used a V0101 vacuum system (Memmert, Schwabach, Germany). The vacuum pressure in the system was 5 m at a temperature of 60 °C. Cuboidal kohlrabi samples, arranged in a single layer in Petri dishes, were vacuum dried for 24 h.

### 3.4. VOC Extraction and Analysis

#### 3.4.1. Methods

Volatile compounds were extracted using the HS-SPME (Headspace Solid Phase Microextraction) method. For this procedure, 200 mg of dried kohlrabi material and 200 mg of homogenized fresh kohlrabi were weighed and transferred into 20 mL headspace vials. To each vial, 50 µL of 2-undecanone (Sigma-Aldrich, Steinheim, Germany) at a concentration of 0.1 mg/mL was added as an internal standard. Additionally, 200 µL of distilled water was added to the vials. Extraction was performed using a 2 cm DVB/CAR/PDMS fiber (Supelco, Bellefonte, PA, USA), pre-conditioned at 250 °C for 5 min. During this time, the samples were incubated at 60 °C for 5 min. The extraction itself was then carried out at 60 °C for 10 min, followed by thermal desorption in the GC injection port for 3 min at 250 °C.

Compound separation and identification were conducted using a Shimadzu QP 2020 Plus gas chromatograph (Shimadzu, Kyoto, Japan), equipped with a ZB-5Msi column (Phenomenex, Torrance, CA, USA), measuring 30 m × 0.25 mm × 0.25 µm. The injector temperature was maintained at 250 °C. Helium served as the carrier gas at a flow rate of 1.0 mL/min, with a linear velocity of 36.3 cm/s and a split ratio of 10. The oven temperature program started at 50 °C, ramped up to 130 °C at 3 °C/min, then to 180 °C at 10 °C/min, and finally reached 280 °C at 20 °C/min. All analyses were performed in triplicate [26,41].

#### 3.4.2. Identification

The identification of volatile compounds was carried out by comparing the obtained mass spectra with reference spectra from the NIST 20 (National Institute of Standards and Technology) and FFNSC (Mass Spectra of Flavors and Fragrances of Natural and Synthetic Compounds) libraries. Additionally, calculated linear retention indices (LRI) were verified using a retention index calculator and matched with values reported in the NIST 20 and FFNSC databases. Further data processing and compound identification were supported using the AMDIS software (v. 2.73) and GCMS Postrun Analysis (Shimadzu, Kyoto, Japan). For additional confirmation, the reference work Identification of Essential Oil Components by Gas Chromatography/Mass Spectrometry (4.1 ed.) by Dr. Robert P. Adams (Biology Department, Baylor University) was consulted [53].

### 3.5. Total Phenolic Content (TPC) and Antioxidant Capacity

For extraction, 0.5 g of kohlrabi powder was mixed with 10 mL of 80% methanol acidified with 1% HCl solution (*v*/*v*). The process was carried out for 20 min in an ultrasonic bath (300 W, 40 kHz; Sonic 6D, Polsonic, Warsaw, Poland), with periodic mixing. This procedure enabled effective separation of the components. After extraction, the suspension was centrifuged at 19,000× *g* for 10 min, and the resulting supernatant was filtered through a hydrophilic PTFE membrane with pores of 0.20 µm (Millex Samplicity™ filter, Merck, Darmstadt, Germany) and used for further analysis.

Total phenolic compounds (TP) were determined using a modified procedure according to the method described by Gao et al. [54]. Briefly, 5 µL of kohlrabi methanol extract was mixed with 50 µL of 10% sodium carbonate solution, 100 µL of distilled water, and 10 µL of Folin–Ciocalteu reagent. After shaking thoroughly (30 s), the samples were incubated for 60 min at room temperature. The absorbance of the mixture was read at 765 nm using a Synergy H1 spectrophotometer (BioTek, Winooski, VT, USA). The results were expressed as milligrams of gallic acid equivalent (GAE) per gram of dry weight (DM) of sample. The measurement was performed in triplicate.

Total antioxidant capacity of the samples was determined using the FRAP assay according to the procedure described by Benzie et al. [55], with modifications. Briefly, 10 μL of kohlrabi methanol extract and 200 μL of freshly prepared FRAP solution were added to the reaction. After 30 s of shaking, the samples were incubated for 10 min, and then absorbance was measured at 593 nm. The antioxidant activity by the DPPH and ABTS methods was assessed according to the protocols described by Yen et al. [56] and Re et al. [57], respectively, with minor modifications, using a Synergy H1 spectrophotometer (BioTek, Winooski, VT, USA). For the DPPH assay, 10 μL of methanol extract was mixed with 200 μL of DPPH solution, and after 10 min of incubation, absorbance was measured at 517 nm. For the ABTS assay, 200 μL of ABTS solution was added to 10 μL of extract, the sample was mixed and incubated for 10 min, and absorbance was measured at 734 nm. For all tests, a calibration curve was developed based on increasing trolox concentrations. Results were expressed as trolox equivalent (Tx) per 100 g of kohlrabi dry weight (μmol Tx/100 g DM).

### 3.6. Water Activity (AW)

Water activity, which measures the amount of free, chemically unbound water in a given material and is available to microorganisms and chemical reactions, is a good indicator of the degree of drying of biological materials. The AquaLab CX-2 meter (AquaLab Dew Point Water Activity Meter 4TE, AquaLab, Warsaw, Poland) was used in this study. Water activity was determined under controlled temperature conditions at 25° C according to the procedure described in the device manufacturer’s specifications. Measurements were performed in four replicates.

### 3.7. Dry Matter (DM)

The weight of fresh, impregnated, and dried kohlrabi was measured using a Radwag AS/160/C/2 scale (Radom, Poland) with a measurement accuracy of ±0.0001 g. The weight of empty Petri dishes was measured, followed by plates containing fresh or vacuum-impregnated material. Finally, the dried weight was determined. The dry weight of both vacuum-impregnated and dried kohlrabi was compared to the dry weight of the raw material—the result was expressed as a percentage. The final result was the arithmetic mean of five measurements.

### 3.8. Color

Color analysis was performed for raw kohlrabi, kohlrabi vacuum-impregnated with two types of impregnating agent, and dried kohlrabi. A Minolta Konica CR-200 colorimeter (Japan) was used. The data were expressed in the CIE L*a*b* system. The L* index value describes the material’s brightness, a* indicates the transition from green to red, and b* indicates the transition from blue to yellow. For each version of the test material, color measurements were taken at ten different locations on the kohlrabi surface. The result was analyzed as the arithmetic mean of the ten measurements. In addition, ∆E was calculated according to the formula given by Li et al. [58].

### 3.9. Density (ρ)

Density analysis of the raw materials was performed using a glass measuring cylinder and a Radwag AS/160/C/2 measuring scale (Radom, Poland) with a precision of ±0.0001 g. The measurement process involved taring the measuring cylinder on the scale, adding a material sample, and then reading its mass and volume. The result was the average of six measurements.

Density (*p*) was calculated using the formula [59]:

(1) $$\rho =\frac{m}{V}\;(\mathrm{kg}/m^{3})$$

where

*m*—material mass [kg];

*V*—material volume [m^3^].

### 3.10. The Volumetric Gelation Index (VGI)

The volumetric gelation index was calculated according to the methodology presented in our previous studies and was calculated using the formula [26]:

(2) $$VGI=\frac{V_{g}}{V_{t}}\;\times 100\;[\%]$$

where

*VGI*—volumetric gel index [%];

*Vg*—volume of gel [mL];

*Vt*—sample volume [mL].

### 3.11. Texture Profile Analysis (TPA)

Texture profile analysis (TPA) is a method for assessing the texture of food subjected to a double-compression test. It provides an objective assessment of sensory characteristics such as hardness, cohesiveness, springiness, and chewiness. This test is widely used in the food industry to assess the quality of food products. The advantage of this method is the use of instrumental analytical tools to describe sensory characteristics, which translates into measurement repeatability, reduced testing costs, and the elimination of subjective sensory perceptions. An Instron 5566 (Instron Corporation, Canton, MA, USA) was used for the analysis. Cuboidal samples were placed on the device base, centrally under the compression plate. For each type of material, control tests are necessary to properly select the sampling time for individual compression parameters (force and deformation). The deformation rate was 0.1 mm s^−1^. The compression process was continued until the sample deformation was half its initial height. Hardness and gumminess were expressed in units of force (N), elasticity in units of strain (mm), and cohesion in units of work (mJ). The compression process was repeated twice to mimic the human chewing process [60]. The measurement was performed in eight repetitions.

### 3.12. Statistical Analysis

Statistical analyses were performed using Statistica version 13.3 (StatSoft, Kraków, Polska). One-way analysis of variance (ANOVA) using Duncan’s test was used to compare the mean values. Differences were considered to be significant at *p* < 0.05. Statistical analyses were performed using R software (R Core Team, 2024). Prior to analysis, the numerical data were standardized. To explore similarities and differences in the composition of major volatile organic compounds (VOCs), hierarchical cluster analysis (HCA) was applied using Ward’s method and Euclidean distance. The results allowed the classification of the samples into three distinct groups. Additionally, principal component analysis (PCA) was performed to determine the relationships between physicochemical properties of kohlrabi and the drying method and vacuum impregnation process.

## 4. Conclusions

The aim of this study was to use vacuum impregnation (VI) for the first time to modify kohlrabi tissue. Vegetable juices (onion juice and beetroot juice) were used as impregnating solutions in the VI process. The dried products were prepared using freeze-drying (FD) and vacuum drying (VD).

The addition of onion juice and beetroot juice significantly modified the volatile compound profile. A total of 34 VOCs were identified. Fifteen VOCs were identified in fresh kohlrabi, the most dominant of which were as follows: 1-Heptene, 2-methyl- (19.81%), Pentyl formate (19.81%), and 4-(Methylthio)butyl isothiocyanate (18.22%). In kohlrabi with onion juice, 21 VOCs were identified, namely dimethyl trisulfide, methyl prop(en)yl disulfide, and 3,5-diethyl-1,2,4-trithiolane, while in kohlrabi with beetroot juice, 13 VOCs were identified, i.e., dimethyl trisulfide, 2-Mercaptoethyl ether, myrcene (0.89–2.48%). FD products retained volatile compounds better than vacuum-dried products. These results confirm that both the choice of vegetable juices and the preservation technology are effective tools for shaping the volatile properties of kohlrabi-based products.

The study showed that vacuum impregnation resulted in higher TPC, antioxidant capacity, dry matter content, water activity, bulk density, volume gel index, color attributes a* and b*, color difference, hardness, and springiness. VI reduced the lightness and consistency of kohlrabi. The values of color attributes and color difference significantly depended on the drying method and the impregnation solution used.

Dried products obtained by the freeze-drying method were characterized by higher TPC, antioxidant capacity, dry matter content, water activity, density, and texture. In addition, freeze-drying allowed for significant preservation of volatile compounds and color of kohlrabi. The studies confirm that FD is the preferred method of food preservation. The results presented in this publication indicate the potential of VI as a method for modifying the properties of kohlrabi and producing functional and innovative dried products.

## 5. Patents

Bogdan, Stępień; Radosław, Maślankowski; Leszek, Rydzak; Marta, Pasławska. A vacuum impregnating machine and method for the initial processing of materials. Wrocław University of Environmental and Life Sciences, Wrocław, PL. Patent Poland No. 421913. 14.06.2017.

## Figures and Tables

**Table 1:** HS-SPME Arrow VOCs profile for fresh and dried kohlrabi.

	Compounds	RT	LRI exp ^1^	LRI lit ^2^	Match ^3^	K_FD %	K_FD SD ^4^	K_VD %	K_VD SD	K %	K SD	KO_FD %	KO_FD SD	KO%	KO SD	KO_VD %	KO_VD SD	KB_FD %	KB_FD SD	KB_VD %	KB_VD SD	KB %	KB SD
**1**	2-Methyl-1-heptene	2.924	784	784	85	8.84 ^ab^	0.29	12.23 ^ab^	0.56	19.81 ^a^	0.20	nd ^5^	0.00	nd	0.00	nd	0.00	nd	0.00	nd	0.00	nd	0.00
**2**	Pentyl formate	4.042	815	823	90	6.10 ^b^	0.04	14.68 ^a^	0.59	0.21 ^c^	0.01	nd	0.00	nd	0.00	nd	0.00	nd	0.00	nd	0.00	nd	0.00
**3**	2-Ethylcyclobutanol	4.626	828	831	85	1.14 ^b^	0.13	6.19 ^a^	0.75	0.17 ^a^	0.01	nd	0.00	nd	0.00	nd	0.00	nd	0.00	nd	0.00	nd	0.00
**4**	3-Methylthio-propionaldehyde	7.176	904	909	93	2.89 ^a^	0.48	0.57 ^b^	0.12	0.02 ^b^	0.01	nd	0.00	nd	0.00	nd	0.00	nd	0.00	nd	0.00	nd	0.00
**5**	(1Z)-1-Propenyl methyl disulfide	8.125	927	928	90	nd	0.00	nd	0.00	nd	0.00	0.39	0.30	0.40	0.11	0.20	0.04	nd	0.00	nd	0.00	nd	0.00
**6**	α-Pinene	8.305	932	933	93	nd	0.00	nd	0.00	nd	0.00	0.23	0.06	0.24	0.13	0.62	0.12	nd	0.00	nd	0.00	nd	0.00
**7**	(E)-1-Propenyl methyl disulfide	8.417	935	928	94	nd	0.00	nd	0.00	nd	0.00	0.69	0.29	1.18	0.40	0.45	0.03	nd	0.00	nd	0.00	nd	0.00
**8**	Camphene	8.887	948	953	97	nd	0.00	nd	0.00	nd	0.00	0.34	0.09	0.07	0.07	0.96	0.03	nd	0.00	nd	0.00	nd	0.00
**9**	Benzaldehyde	7.291	958	960	96	2.92 ^a^	0.55	2.52 ^a^	0.29	0.65 ^a^	0.01	nd	0.00	nd	0.00	nd	0.00	nd	0.00	nd	0.00	nd	0.00
**10**	Dimethyl trisulfide	9.251	965	972	95	0.87 ^b^	0.62	6.58 ^b^	0.84	0.34 ^b^	0.07	17.17 ^b^	1.28	71.21 ^a^	1.98	16.00 ^b^	1.01	53.59 ^a^	1.10	10.14 ^b^	0.27	61.07 a	0.88
**11**	1-Octen-3-ol	9.516	969	977	93	38.30 ^b^	1.79	19.37 ^bc^	0.95	60.03 ^a^	0.37	nd	0.00	nd	0.00	nd	0.00	nd	0.00	nd	0.00	nd	0.00
**12**	6-Methyl-hept-5-en-2-one	10.095	981	986	90	nd	0.00	nd	0.00	nd	0.00	1.00 ^b^	0.12	0.14 ^b^	0.16	3.83 ^a^	0.01	nd	0.00	nd	0.00	nd	0.00
**13**	Myrcene	10.329	988	991	95	0.76 ^bc^	0.44	3.66 ^bc^	0.01	0.05 ^c^	0.01	13.40 ^bc^	1.04	0.12 ^c^	0.05	36.46 ^a^	0.07	2.48 ^bc^	0.16	21.71 ^ab^	1.72	0.89 ^bc^	0.07
**14**	unknown	10.329	993			35.08 ^a^	1.42	31.05 ^ab^	0.48	1.13 ^b^	0.26	nd	0.00	nd	0.00	nd	0.00	nd	0.00	nd	0.00	nd	0.05
**15**	*p*-Mentha-1(7),8-diene	10.917	1003	1004	97	nd	0.00	nd	0.00	nd	0.00	0.70	0.36	0.04	0.01	1.99	0.02	nd	0.00	nd	0.00	nd	0.00
**16**	unknown	11.023	1006			nd	0.00	nd	0.00	nd	0.00	6.42 ^a^	0.30	1.86 ^a^	0.13	2.04 ^a^	0.04	1.81 ^a^	0.19	0.38 ^a^	0.03	0.36 ^a^	0.05
**17**	*p*-Cymene	11.75	1022	1035	92	nd	0.00	nd	0.00	nd	0.00	0.45	0.24	0.05	0.03	1.28	0.02	nd	0.00	nd	0.00	nd	0.00
**18**	2-Ethylhexanol	11.893	1026	1030	93	8.44 ^a^	0.83	3.01 ^a^	0.71	2.89 ^a^	0.28	3.66 ^a^	0.21	0.49 ^a^	0.08	2.01 ^a^	0.04	1.39 ^a^	0.34	1.20 ^a^	0.06	1.83 ^a^	0.19
**19**	unknown	11.912	1031			1.44	0.20	2.01	0.21	0.24	0.05	nd	0.00	nd	0.00	nd	0.00	nd	0.00	nd	0.00	nd	0.00
**20**	unknown	14.073	1076			nd	0.00	nd	0.00	nd	0.00	nd	0.00	nd	0.00	nd	0.00	20.61 ^b^	1.43	63.25 ^a^	1.50	5.57 ^bc^	0.21
**21**	*n*-Nonanal	15.196	1102	1107	85	0.30	0.04	0.45	0.45	0.10	0.02	0.33	0.21	0.10	0.01	0.45	0.07	0.55	0.15	0.90	0.01	0.52	0.06
**22**	2-Mercaptoethyl ether	15.199	1122		85	0.97 ^b^	0.24	1.16 ^b^	0.11	0.06 ^b^	0.03	3.42 ^b^	0.62	2.18 ^b^	0.11	1.88 ^b^	0.12	3.34 ^b^	0.13	1.95 ^b^	0.20	14.11 ^a^	0.10
**23**	Menthone	16.874	1153	1148	92	nd	0.00	nd	0.00	nd	0.00	43.88 ^a^	0.71	6.59 ^b^	0.41	23.09 ^ab^	1.04	nd	0.00	nd	0.00	nd	0.00
**24**	(1E)-1-Propenyl methyl trisulfide	17.66	1159	1179	90	nd	0.00	nd	0.00	nd	0.00	0.19	0.13	0.20	0.01	0.14	0.05	nd	0.00	nd	0.00	nd	0.00
**25**	(1Z)-1-Propenyl methyl trisulfide	17.896	1161	1179	87	nd	0.00	nd	0.00	nd	0.00	0.28	0.19	0.26	0.09	0.15	0.06	nd	0.00	nd	0.00	nd	0.00
**26**	(1,1-Dimethylethyl)(1-methylpropyl) disulfide	19.033	1185	1169	85	nd	0.00	nd	0.00	nd	0.00	nd	0.00	nd	0.00	nd	0.00	3.23 ^a^	0.18	0.15 ^b^	0.01	0.09 ^b^	0.01
**27**	Methyl tetrasulfide	20.157	1210	1214	93	1.38 ^c^	0.22	0.34 ^c^	0.06	16.16 ^a^	0.09	4.56 ^bc^	0.46	11.85 ^ab^	0.30	1.98 ^c^	0.13	1.78 ^c^	0.21	0.07 ^c^	0.01	6.13 ^bc^	0.17
**28**	3,5-Diethyl-1,2,4-trithiolane	25.818	1339	1344	85	nd	0.00	nd	0.00	nd	0.00	0.48	0.09	0.18	0.04	0.23	0.08	nd	0.00	nd	0.00	nd	0.00
**29**	2,5-Dimethylhexane-2,5-dihydroperoxide	26.43	1354	1367	85	nd	0.00	nd	0.00	nd	0.00	nd	0.00	nd	0.00	nd	0.00	9.20 ^a^	1.00	0.11 ^b^	0.05	0.04 ^b^	0.02
**30**	unknown	26.626	1359			nd	0.00	nd	0.00	nd	0.00	1.09 ^b^	0.32	1.52 ^b^	0.35	0.40 ^b^	0.04	1.24 ^b^	0.13	0.07 ^b^	0.05	8.99 ^a^	1.01
**31**	*n*-Tetradecane	28.264	1400	1400	92	nd	0.00	nd	0.00	nd	0.00	nd	0.00	nd	0.00	nd	0.00	0.53	0.24	0.37	0.02	0.18	0.03
**32**	Erucin	28.968	1417	1432	85	nd	0.00	nd	0.00	nd	0.00	1.06 ^b^	0.25	1.41 ^ab^	0.05	5.16 ^a^	1.05	nd	0.00	nd	0.00	nd	0.00
**33**	4-(Methylthio)butyl isothiocyanate	28.996	1418	1433	90	0.72 ^b^	0.23	5.92	0.16	18.22 ^a^	0.25	nd	0.00	nd	0.00	nd	0.00	0.49 ^b^	0.10	0.16 ^b^	0.13	0.31 ^b^	0.05
**34**	Phenethyl isothiocyanate	29.831	1439	1454	90	nd	0.00	nd	0.00	nd	0.00	0.61	0.36	0.17	0.03	0.98	0.11	nd	0.00	nd	0.00	nd	0.00

^1^ LRI exp—experimentally calculated LRI; ^2^ LRI lit—LRI available in library; ^3^ Mass spectra similarity match [%], ^4^ standard deviation; ^5^ nd—not detected; K—kohlrabi; KO—kohlrabi with onion juice; KB—kohlrabi with beetroot juice; FD—freeze-drying, VD—vacuum drying. The values marked with different lowercase letters (a–c) indicate significant differences (*p* < 0.05).

**Table 2:** Color parameters of raw and dried kohlrabi: L*—lightness; a*—(+) redness/(−) greenness; b*—(+) yellowing; ΔE—total color of vegetables.

Method	L*	a*	b*	∆E
K	79.98 ± 0.94 ^d^	−0.91 ± 0.35 ^a^	7.94 ± 0.82 ^a^	-
K_FD	92.50 ± 1.52 ^e^	−0.48 ± 0.16 ^a^	11.36 ± 0.52 ^b^	12.98
K_VD	74.27 ± 4.13 ^c^	0.47 ± 0.86 ^a^	22.96 ± 2.52 ^d^	16.13
KO	73.41 ± 3.72 ^c^	−1.52 ± 0.31 ^a^	9.00 ± 0.71 ^a^	6.69
KO_FD	91.23 ± 3.69 ^e^	−1.01 ± 0.14 ^a^	11.90 ± 1.01 ^b^	11.93
KO_VD	66.04 ± 5.81 ^b^	9.65 ± 1.92 ^b^	31.68 ± 2.50 ^f^	29.49
KB	64.42 ± 4.59 ^b^	14.36 ± 3.75 ^c^	11.27 ± 1.86 ^b^	22.06
KB_FD	75.72 ± 3.08 ^c^	13.90 ± 3.47 ^c^	17.51 ± 2.28 ^c^	18.13
KB_VD	51.43 ± 4.30 ^a^	13.32 ± 1.50 ^c^	25.22 ± 3.33 ^e^	36.28

The values (mean of three replications) ± standard deviation followed by different letters (a–f) are different (*p* ≤ 0.05) according to Duncan’s test. K: kohlrabi; KO: kohlrabi with onion juice; KB: kohlrabi with beetroot juice; FD: freeze-drying; VD: vacuum drying.

**Table 3:** Total phenolic content (mg GAE/100 g dry matter) and antioxidant capacity (µMol Trolox/100 g of dry matter) of kohlrabi.

(mg/g DM)	TPC	ABTS	FRAP	DPPH
K	563.83 ± 1.23 ^c^	1786.38 ± 6.16 ^bc^	1625.71 ± 11.75 ^e^	339.51 ± 4.58 ^e^
K_FD	550.13 ± 2.58 ^c^	1752.78 ± 5.78 ^b^	1401.02 ± 12.12 ^c^	153.27 ± 6.28 ^a^
K_VD	421.48 ± 0.96 ^a^	1636.02 ± 11.08 ^a^	1309.80 ± 7.99 ^a^	143.35 ± 4.29 ^a^
KO	603.62 ± 3.13 ^d^	2148.57 ± 7.82 ^f^	1958.02 ± 10.53 ^i^	508.46 ± 3.55 ^h^
KO_FD	958.59 ± 5.28 ^f^	1895.03 ± 10.56 ^d^	1841.85 ± 16.11 ^h^	459.25 ± 3.69 ^g^
KO_VD	477.22 ± 3.47 ^b^	1796.02 ± 4.78 ^c^	1463.00 ± 10.85 ^d^	300.90 ± 5.22 ^d^
KB	588.12 ± 6.12 ^d^	2030.60 ± 14.51 ^e^	1782.90 ± 13.54 ^g^	373.35 ± 5.08 ^f^
KB_FD	679.89 ± 3.88 ^e^	1803.21 ± 16.68 ^c^	1744.189 ± 12.78 ^f^	200.74 ± 4.39 ^c^
KB_VD	461.48 ± 2.85 ^b^	1755.04 ± 14.09 ^b^	1359.80 ± 14.52 ^b^	171.28 ± 3.07 ^b^

The values (mean of three replications) ± standard deviation followed by different letters (a–i) are different (*p* ≤ 0.05) according to Duncan’s test. K: kohlrabi; KO: kohlrabi with onion juice; KB: kohlrabi with beetroot juice; FD: freeze-drying; VD: vacuum drying.

**Table 4:** The explanation of the samples coding.

Code	Material	Type of Drying
K	Kohlrabi	-
K_FD	Kohlrabi	freeze-drying
K_VD	Kohlrabi	vacuum drying
KO	Kohlrabi with onion juice	-
KO_FD	Kohlrabi with onion juice	freeze-drying
KO_VD	Kohlrabi with onion juice	vacuum drying
KB	Kohlrabi with beetroot juice	-
KB_FD	Kohlrabi with beetroot juice	freeze-drying
KB_VD	Kohlrabi with beetroot juice	vacuum drying

## Data Availability

Data are contained within the article.
